# STRESS, FAITH, AND HEALTH AMONG BLACK MEN IN MIDDLE AND LATE LIFE

**DOI:** 10.1093/geroni/igz038.782

**Published:** 2019-11-08

**Authors:** Marino A Bruce, Roland J Thorpe

**Affiliations:** 1 Vanderbilt University, Nashville, Tennessee, United States; 2 Johns Hopkins Bloomberg School of Public Health, Baltimore, Maryland, United States

## Abstract

African American men experience extremely high levels of social and psychological stress from unfavorable social and economic circumstances emerging from institutional discrimination and unfair treatment. Stress has been linked to disproportionate risks for illness, disease and premature mortality among this population. But, few studies have examined how African American men manage or cope with stress, and even fewer have assessed how their coping responses have implications for their health. Faith has been considered a stress coping strategy and a growing number of studies explore how religiosity and spirituality have implications for health outcomes. No studies to our knowledge have examined how faith impacts stress and its influence on the health among African American men. The purpose of this chapter is to demonstrate how faith has implications for socio-biologic interactions associated with elevated risk for disease and premature death among this marginalized population.

